# Genetic association of TLR4 Asp299Gly, TLR4 Thr399Ile, and CD14 C‐159T polymorphisms with the risk of severe RSV infection: a meta‐analysis

**DOI:** 10.1111/irv.12378

**Published:** 2016-03-07

**Authors:** Jiahui Zhou, Xiangning Zhang, Shuming Liu, Ziyou Wang, Qicong Chen, Yongfu Wu, Zhiwei He, Zunnan Huang

**Affiliations:** ^1^ China‐America Cancer Research InstituteDongguan Scientific Research CenterGuangdong Medical UniversityDongguanGuangdongChina; ^2^ Key Laboratory for Medical Molecular Diagnostics of Guangdong ProvinceDongguanGuangdongChina; ^3^ Department of PathophysiologyGuangdong Medical UniversityDongguanGuangdongChina; ^4^ School of Preclinical MedicineGuangxi Medical UniversityNanningGuangxiChina; ^5^ Affiliated Hospital of Guangdong Medical UniversityZhanjiangGuangdongChina

**Keywords:** Asp299Gly, C‐159T, CD14, meta‐analysis, polymorphisms, RSV infection, Thr399Ile, TLR4

## Abstract

Respiratory syncytial virus (RSV) is the most frequent cause of hospitalization in infants worldwide. It is recognized by Toll‐like receptor 4 (TLR 4) and cluster of differentiation 14 (CD14) in the innate immune response. Previous case–control studies reported the influence of TLR4 Asp299Gly, TLR4 Thr399Ile, and CD14 C‐159T polymorphisms on the risk of severe RSV infection. However, a decisive conclusion has not been achieved. Therefore, we performed this meta‐analysis to examine the association between these three polymorphisms and the development of RSV bronchiolitis. A systematic literature search was performed using the PubMed, EMbase, Google Scholar Search, China National Knowledge Infrastructure, China Biological Medicine, and Wanfang Databases. The data were extracted and pooled odds ratios with 95% confidence intervals were calculated under six genetic models. A total of six studies with 1009 cases and 1348 controls, three studies with 473 cases and 481 controls, or four studies with 325 cases and 650 controls relating to each of the three polymorphisms were included in this meta‐analysis. The analyzed data indicated that all of these polymorphisms were not associated with the risk of severe RSV infection. This is the first meta‐analysis to investigate the relationship of TLR4 Asp299Gly, TLR4 Thr399Ile, and CD14 C‐159T polymorphisms with the risk of severe RSV infection. Although the results of this retrospective analysis indicated a lack of the association, more extensive multicentric studies with large sample sizes are necessary to provide a more reliable estimation of the association between these three polymorphisms and RSV bronchiolitis susceptibility.

## Introduction

Respiratory syncytial virus (RSV) is a member of paramyxoviridae family. It is an RNA virus which could cause serious lower respiratory tract infection in children.^1^, ^2^ RSV is also recognized as an important cause for severe respiratory disease in the elderly and immune‐compromised populations. Up to date, there is still no specific treatment or efficient vaccine to combat RSV. As a result, RSV contributes to the global burden of disease worldwide.^3^ Although great efforts have been made to study the mechanisms underlying differential response to RSV, the pathogenesis of RSV infection still remains unknown.^1^

Whether individuals are susceptible to RSV and how they respond to RSV are determined by the comprehensive factors rather than a single factor. Environmental factors, such as PM10 (particular matter less than 10 μm in aerodynamic diameter) exposure or cigarette smoke exposure, contribute to susceptibility and response to RSV infection.^4^, ^5^ Other factors including age, race, maternal atopy, and genetic polymorphisms are the risk factors as well.^5^, ^6^, ^7^

It has been reported that genetic factors might influence the susceptibility to RSV infection in early life.^8^ In this article, we focused on the association of the TLR4 (Toll‐like receptor 4) and CD14 (cluster of differentiation 14) with severe RSV infection. TLR4 belongs to the mammalian Toll‐like receptor family, which play an important role in regulating innate immunity to a variety of microbes including RSV.^9^ CD14 is a lipopolysaccharide (LPS) receptor, which is necessary for initiating the innate response to LPS.^10^ CD14 is assumed to form a complex with TLR4–MD2 (MD2 also known as lymphocyte antigen 96). In addition, CD14 appears to function as ‘caretaker’ for activating TLR4‐dependent pathway.^11^ Thus, both TLR4 and CD14 genes may participate in severe RSV infection.

Several publications have reported the association of single‐nucleotide polymorphisms (SNPs) of Asp299Gly, Thr399Ile in TLR4 and C‐159T in CD14 with the risk of RSV infection, but the conclusions were inconsistent. What is more, no meta‐analysis has been conducted to investigate the association between these three polymorphisms and the risk of RSV infection so far. Therefore, we performed this meta‐analysis to estimate the potential relevance of TLR4 Asp299Gly, TLR4 Thr399Ile, and CD14 C‐159T polymorphisms with the susceptibility of severe RSV infection.

## Materials and methods

The present meta‐analysis was conducted according to the Preferred Reporting Items for Systematic Reviews and Meta‐Analyses (PRISMA) statement,^12^ and a completed PRISMA checklist is shown in Table S1 PRISMA 2009 checklist of the Supporting Information. In addition, this study also followed some guidelines proposed by the Human Genome Epidemiology Network for systematic review of genetic association studies.^13^

### Study identification and selection

We carried out a comprehensive and systematic literature search based on the PubMed, EMbase, Google Scholar Search, China National Knowledge Infrastructure (CNKI), China Biological Medicine (CBM), and Wanfang Databases. In order to identify the related articles as many as possible, we used the keywords including ‘RSV or respiratory syncytial virus and TLR4 or Toll‐like receptor 4 or CD14 and polymorphism or polymorphisms or variant or mutation’ to obtain all genetic studies on the relationship of single‐nucleotide polymorphisms (SNPs) in the TLR4 and CD14 genes with severe RSV infection. We also conducted a manual search of references of original or reviewed articles on this subject to identify additional studies. The searches were last updated on June 23, 2015.

### Inclusion and exclusion criteria

Eligible studies were selected according to the following inclusion criteria: (i) on the association of TLR4 Asp299Gly (rs4986790), TLR4 Thr399Ile (rs4986791), or CD14 C‐159T (rs2569190) polymorphisms with severe RSV infection; (ii) in a case–control design; (iii) with useful genotype frequencies. The exclusion criteria were (i) animal studies; (ii) abstracts, editorials, and review articles; (iii) studies without sufficient genotype data. The included studies were restricted to English and Chinese. Only one could be accepted if the publications were duplicated. Two reviewers extracted eligible studies independently according to the inclusion and exclusion criteria, and any disagreement was resolved by discussion among the authors.

### Data extraction

One author extracted the following information from each eligible study: name of the first author, year of publication, the country and ethnicity of the participants, the number of RSV cases and controls, the number of genotypes of TLR4 (Asp299Gly and Thr399Ile) and CD14 (C‐159T) polymorphism in RSV cases and controls, Hardy–Weinberg equilibrium (HWE), and genotyping method. Then, another author checked the data carefully to ensure that they are complete and correct. We did not define a minimum number of patients to be included in our meta‐analysis.

### Statistical analysis

In this meta‐analysis, the pooled odds ratios (ORs) with 95% confidence intervals (CIs) were used to estimate the association of TLR4 Asp299Gly, TLR4 Thr399Ile, and CD14 C‐159T polymorphism with the risk of severe RSV infection. Six genetic models, namely the allelic, homozygous, heterozygous, dominant, recessive, and overdominant models, were performed, respectively. Z‐test was used to assess the significance of the pooled ORs, and *P* ≤ 0·05 was considered as statistically significant. We performed I^2^ test to evaluate between‐study heterogeneity according to the criteria from the Cochrane Handbook,^14^ which categorized it into unimportant (0–40%), moderate (30–60%), substantial (50–90%), and considerable (75–100%). I^2^ statistics was presented together with its 95% confidence intervals.^15^, ^16^ Fixed‐effect model was used to calculate the ORs and 95% CIs of any genetic model without substantial heterogeneity (I^2^ < 50%); otherwise, random‐effect model was selected. Both the Begg and Egger tests were used to assess the publication bias, and a *P* value of less than 0·05 indicated the presence of publication bias. The trim and fill method was also employed to identify and correct funnel plot asymmetry arising from publication bias.^17^ In case–control studies, Hardy–Weinberg equilibrium (HWE) was tested by chi‐square (χ2) to evaluate the study quality of genotype data, and *P* < 0·05 was considered statistically significant. A high‐quality study was said that its control group was in HWE. A study without HWE in controls was defined as a low‐quality one. Low‐quality studies were excluded in the sensitivity analysis. Newcastle–Ottawa Scale (NOS) criteria 18, 19 were used to assess the overall quality of the included studies. The evaluation of content in the NOS was classified into three independent aspects: object selection, comparability, and exposure assessment. A study of high quality should obtain at least five points in the NOS quality assessment. Both HWE and NOS were conducted in our meta‐analysis. Sensitivity analysis was conducted by sequentially excluding individual studies to estimate the stability of the meta‐analysis results. Data analysis was performed using STATA 14.0 software (Stata Corp, College Station, Texas, USA) and Review Manager 5.3 (The Cochrane Collaboration, http://ims.cochrane.org/revman).

## Results

**Figure 1:**
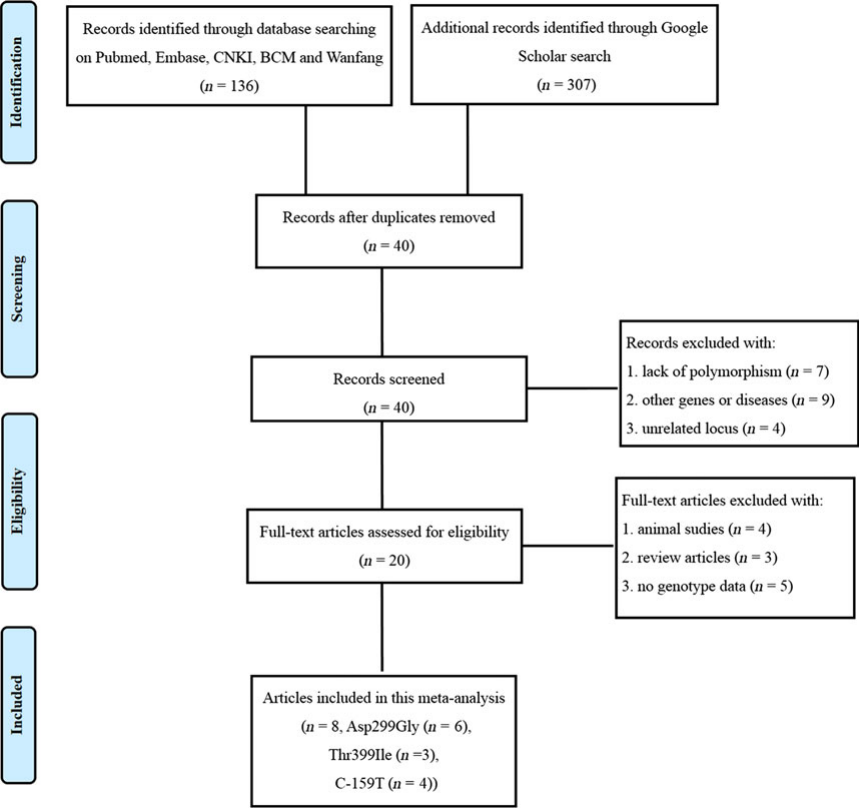
A flow diagram of the process used to select eligible studies.

A flow diagram describing the selection procedure of the eligible studies included in this meta‐analysis is shown in Figure 1. The initial search strategy yielded a total of 443 (= 136 + 307) potential records up to June 23, 2015. Forty records were then obtained after the duplicates were weeded out. After a careful check of their abstracts and/or full‐text reviews, 20 articles were excluded unrelated to TLR4 Asp299Gly (rs4986790), TLR4 Thr399Ile (rs4986791), CD14 C‐159T (rs2569190) polymorphisms and RSV bronchiolitis. According to the exclusion criteria, 12 articles were further discarded. Finally, we obtained eight eligible articles for our meta‐analysis, which included six studies related to TLR4 Asp299Gly polymorphism, three studies linked to TLR4 Thr399Ile polymorphism, and four studies connected to CD14 C‐159T polymorphism. The main characteristics of all these studies are listed in Table 1. The genotype distributions of all 13 studies in the control groups conformed to the HWE except for one reported by Goutaki *et al*.^20^ (Table 1). This study was also included in the meta‐analysis, but excluded in the sensitivity analysis. The NOS results showed that the quality score of any included study ranged from six to nine. Thus, all the studies included in our meta‐analysis were of high quality (Table 2).

**Table 1:** The baseline characteristics of all the studies included in the meta‐analysis

Gene locus	SNP (Gene)	Study	Area	Ethnicity	No. of cases	No. of controls	Genotype						HWE P	Genotyping method
							case			control				
rs4986790	Asp299Gly (TLR4)						AA	AG	GG	AA	AG	GG		
		Puthothu *et al*. (2006)^22^	Germany	Caucasian	131	269	126	5	0	245	22	2	0·117	PCR‐RFLP
		Paulus *et al*. (2007)^21^	Britain	Caucasian	136	113	128	7	1	104	9	0	1·000	TaqMan
		Helminen *et al*. (2008)^23^	Finland	Caucasian	97	398	80	17	0	327	66	5	0·384	TaqMan
		Lofgren *et al*. (2010)^24^	Finland	Caucasian	312	356	251	55	6	290	59	7	0·076	PCR‐RFLP
		Kresfelder *et al*. (2011)^25^	South Africa	African	283	113	265	18	0	99	14	0	1·000	TaqMan
		Goutaki *et al*. (2014)^20^	Northern Greece	Caucasian	50	99	45	2	3	93	1	5	0·000	PCR‐RFLP
rs4986791	Thr399Ile (TLR4)						CC	CT	TT	CC	CT	TT		
		Puthothu *et al*. (2006)^22^	Germany	Caucasian	131	269	120	11	0	244	23	2	0·130	PCR‐RFLP
		Kresfelder *et al*. (2011)^25^	South Africa	African	292	113	288	4	0	113	0	0	1·000	PCR‐RFLP
		Goutaki *et al*. (2014)^20^	Northern Greece	Caucasian	50	99	31	12	7	51	35	13	0·099	PCR‐RFLP
rs2569190	C‐159T (CD14)						CC	CT	TT	CC	CT	TT		
		Tal *et al*. (2004)^26^	Finland	Caucasian	99	90	27	60	12	25	53	12	0·084	PCR‐RFLP
		Puthothu *et al*. (2006)^22^	Germany	Caucasian	127	261	41	59	27	79	124	58	0·533	PCR‐RFLP
		Inoue *et al*. (2007)^27^	Japan	Asian	49	203	11	18	20	38	103	62	0·776	PCR‐RFLP
		Goutaki *et al*. (2014)^20^	Northern	Caucasian	50	96	13	16	21	30	46	20	0·099	PCR‐RFLP

**Table 2:** Quality assessment of the included studies based on Newcastle–Ottawa Scale criteria

Literature	Selection				Comparability	Exposure			Total
	I	II	III	IV	V	VI	VII	VIII	
Puthothu *et al*. (2006)	*	*	*	*	*	*	*	*	********
Paulus *et al*. (2007)	*	*	*	*	**	*	*	*	*********
Helmmen *et al*. (2008)	*	*			*	*	*	*	******
Lofgren *et al*. (2010)	*	*	*	*	*	*	*	*	********
Kresfelder *et al*. (2011)	*	*	*	*	*	*	*	*	********
Goutaki *et al*. (2014)	*	*	*	*	**	*	*	*	*********
Tal *et al*. (2004)	*	*	*		*	*		*	******
Inoue *et al*. (2007)	*	*	*		*	*	*	*	*******

I, Is the case definition adequate? II, Representativeness of the cases II, Selection of Controls IV, Definition of Controls V, Comparability of cases and controls on the basis of the design or analysis VI, Ascertainment of exposure VII, Same method of ascertainment for cases and controls VIII, Non‐Response rate.

### A meta‐analysis of TLR4 Asp299Gly polymorphism with the risk of RSV infection

**Figure 2:**
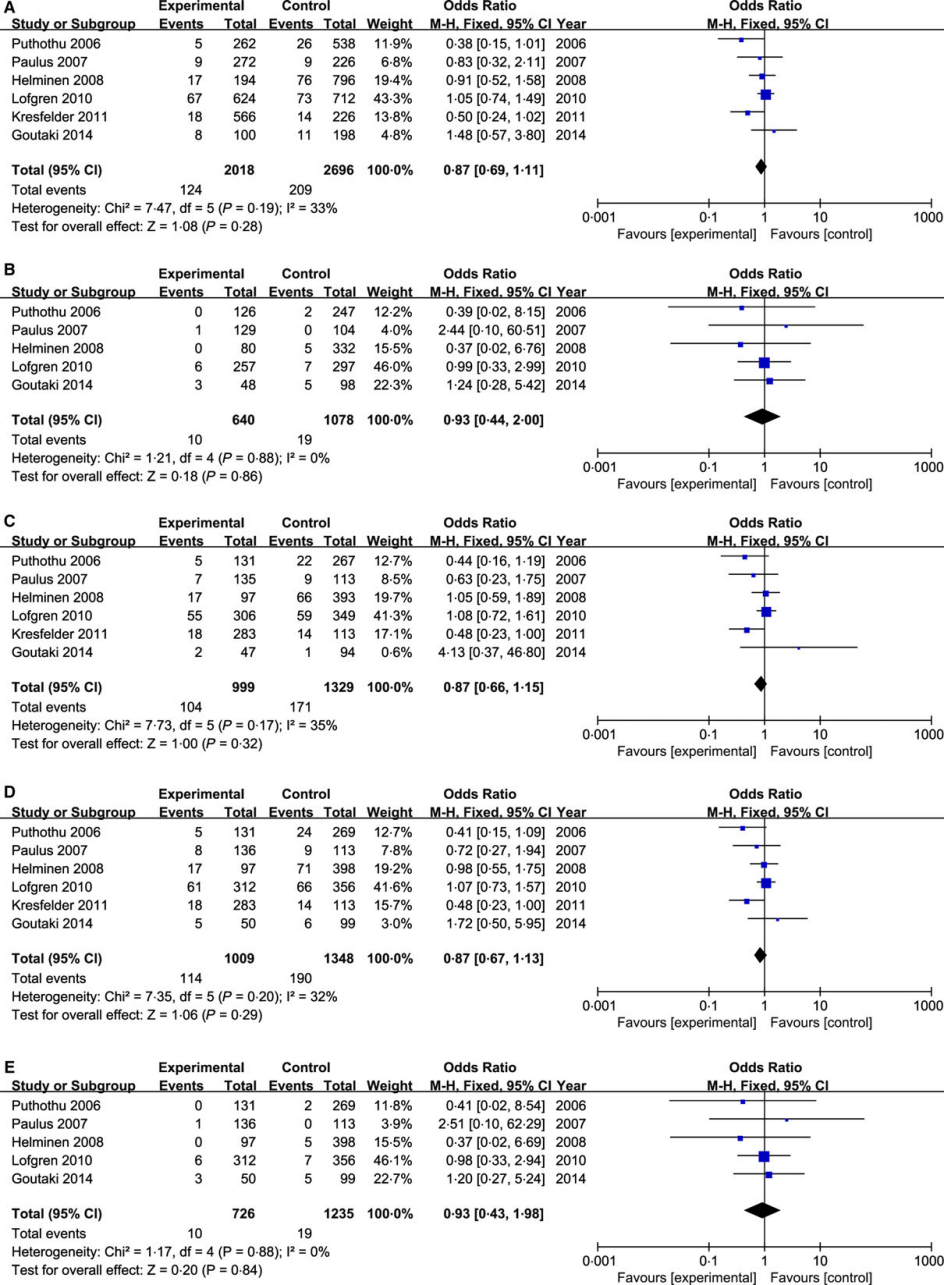
Forest plots of TLR4 Asp299Gly (rs4986790) polymorphism and the risk of severe RSV infection in five genetic models. (A) the allelic model (G versus A); (B) the homozygous model (GG versus AA); (C) the heterozygous model (GA versus AA); (D) the dominant model (GG + GA versus AA); (E) the recessive model (GG versus GA + AA).

In this meta‐analysis, a total of six studies 20, 21, 22, 23, 24, 25 involving 1009 cases and 1348 controls were included to investigate the association between TLR4 Asp299Gly polymorphism and the risk of severe RSV infection (as shown in Table 1). The meta‐analysis showed no substantial heterogeneity among studies in five genetic models (I^2^ < 50%); thus, the fixed‐effect model was used to calculate the pooled ORs of these models. Combined data revealed that TLR4 Asp299Gly polymorphism was not associated with the risk of severe RSV infection in any genetic model (the allelic model: G versus A: OR = 0·87, 95% CI 0·69–1·11, *P* = 0·28; the homozygous model: GG versus AA: OR = 0·93, 95% CI 0·44–2·00, *P* = 0·86; the heterozygous model: GA versus AA: OR = 0·87, 95% CI 0·66–1·15, *P* = 0·32; the dominant model: GG + GA versus AA: OR = 0·87, 95% CI 0·67–1·13, *P* = 0·29; and the recessive model: GG versus GA + AA; OR = 0·93, 95% CI 0·43–1·98, *P* = 0·84) (as shown in Table 3 and Figure 2). We also conducted a subgroup analysis by ethnicity in Caucasian (six studies) and Asian (one study), but no significant difference was found between the general population and two ethnic subgroups (data not shown here).

**Table 3:** Meta‐analysis of the association between three polymorphisms in the TLR4/CD14 genes and risk of severe RSV infection

Gene locus	Genetic comparison	Effect Model	OR(95%CI)	p_or_	I^2^(%) (95%CI)	Begg's test(z, *P*)	Egger's test(t, *P*)
rs4986790	G versus A	Fixed	0·87 (0·69, 1·11)	0·28	33 (0, 0·73)	1·13, 0·26	−1·11, 0·33
rs4986790	GG versus AA	Fixed	0·93 (0·44, 2·00)	0·86	0 (0, 0·79)	0·24, 0·81	−0·59, 0·60
rs4986790	GA versus AA	Fixed	0·87 (0·66, 1·15)	0·32	35 (0, 0·74)	0·00, 1·00	−0·44, 0·68
rs4986790	GG + GA versus AA	Fixed	0·87 (0·67, 1·13)	0·29	32 (0, 0·72)	0·38, 1·07	−0·90, 0·42
rs4986790	GG versus GA + AA	Fixed	0·93 (0·43, 1·98)	0·84	0 (0, 0·79)	0·24, 0·81	−0·53, 0·63
rs4986791	T versus C	Fixed	0·84 (0·55, 1·29)	0·43	0 (0, 0·90)	1·04, 0·30	7·28, 0·09
rs4986791	TT versus CC	Fixed	0·80 (0·31, 2·10)	0·65	0 (0, 0·90)	0·00, 100	−
rs4986791	TC versus CC	Fixed	0·81 (0·48, 1·37)	0·44	0 (‐)	0·00, 1·00	0·94, 0·52
rs4986791	TT + TC versus CC	Fixed	0·81 (0·50, 1·33)	0·40	0 (‐)	1·04, 0·30	1·93, 0·30
rs4986791	TT versus TC + CC	Fixed	0·96 (0·38, 2·42)	0·93	0 (0, 0·90)	0·00, 1·00	−
rs2569190	T versus C	Fixed	1·09 (0·89, 1·32)	0·40	31 (0, 0·75)	1·70, 0·09	1·95, 0·19
rs2569190	TT versus CC	Fixed	1·14 (0·78, 1·68)	0·49	15 (0, 0·61)	0·34, 0·73	0·83, 0·50
rs2569190	TC versus CC	Fixed	0·87 (0·63, 1·22)	0·42	0 (0, 0·85)	0·34, 0·73	−1·07, 0·40
rs2569190	TT + TC versus CC	Fixed	0·97 (0·71, 1·32)	0·85	0 (0, 0·85)	1·04, 0·96	1·04, 0·96
rs2569190	TT versus TC + CC	Random	1·36 (0·83, 2·23)	0·22	53 (0, 0·84)	0·34, 0·73	0·54, 0·65
rs2569190	TC versus TT + CC	Fixed	0·81 (0·61, 1·06)	0·13	30 (0, 0·75)	1·02, 0·308	−1·59, 0·254

### A meta‐analysis between TLR4 Thr399Ile polymorphism and the risk of RSV infection

A meta‐analysis of the association between TLR4 Thr399Ile polymorphism and the risk of severe RSV infection included three studies 20, 22, 25 with a total of 473 cases and 481 controls (as shown in Table 1). Due to unobserved heterogeneity among these three studies (I^2^ = 0%), the fixed‐effect model was used to calculate the pooled ORs in all genetic models. The results showed that no association of TLR4 Thr399Ile polymorphism was observed with the risk of severe RSV infection in five genetic models (the allelic model: T versus C: OR = 0·84, 95% CI 0·55–1·29, *P* = 0·43; the homozygous model: TT versus CC: OR = 0·80, 95% CI 0·31–2·10, *P* = 0·65; the heterozygous model: TC versus CC: OR = 0·81, 95% CI 0·48–1·37, *P* = 0·44; the dominant model: TT + TC versus CC: OR = 0·81, 95% CI 0·50–1·33, *P* = 0·40; and the recessive model: TT versus TC + CC; OR = 0·96, 95% CI 0·38–2·42, *P* = 0·93) (Table 3).

### A meta‐analysis of CD14 C‐159T polymorphism on the risk of RSV infection

**Figure 3:**
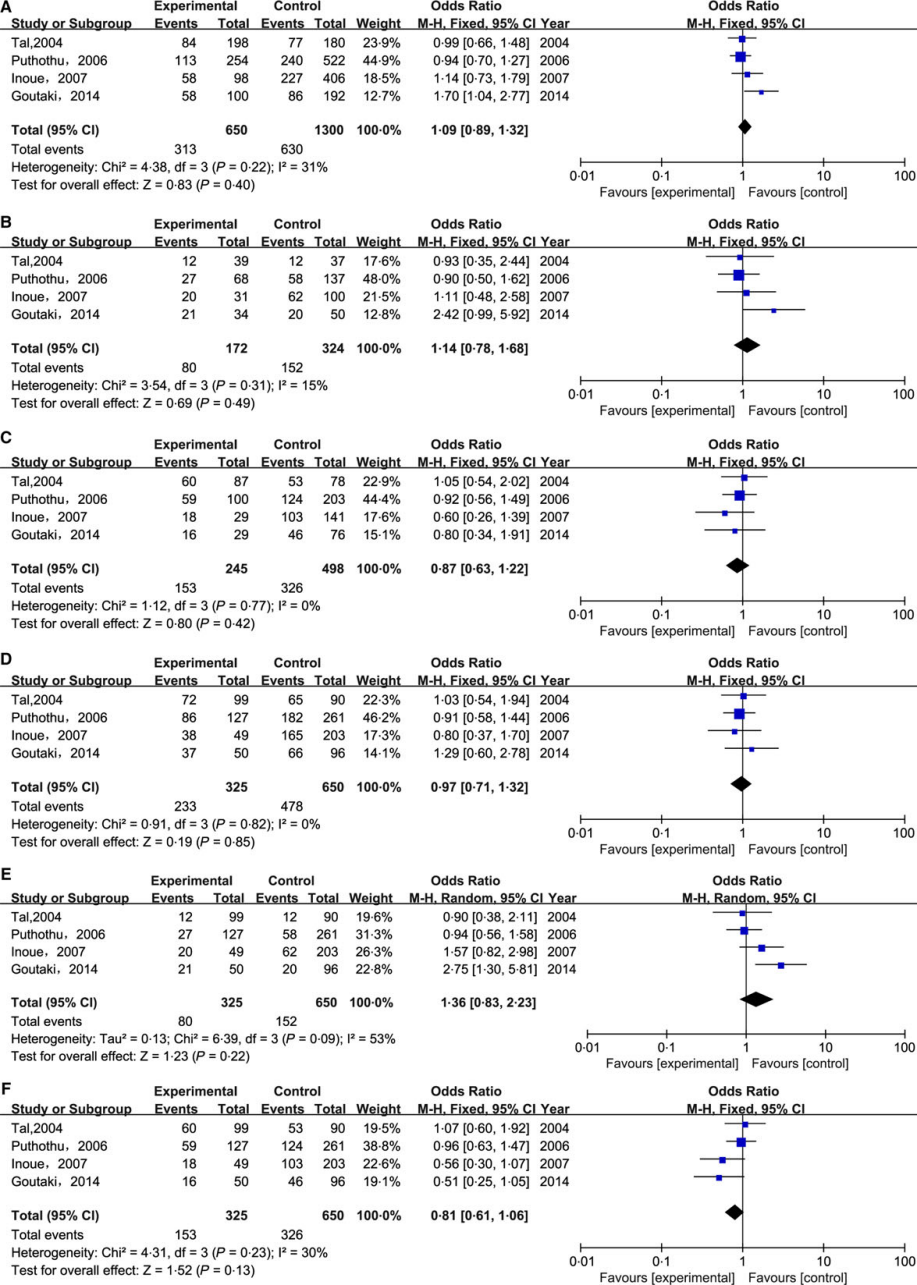
Forest plots of CD14 C‐159T (rs2569190) polymorphism and the risk of severe RSV infection in six genetic models. (A) the allelic model (T versus C); (B) the homozygous model (TT versus CC); (C) the heterozygous model (TC versus CC); (D) the dominant model (TT + TC versus CC); (E) the recessive model (TT versus TC + CC); (F) the overdominant model (TC versus TT + CC).

In this retrospective analysis, four eligible studies 20, 22, 26, 27 involving 325 cases and 650 controls were collected to evaluate the association between TLR4 Asp299Gly polymorphism and the risk of severe RSV infection (as shown in Table 1). Among all the genetic models used here, only the recessive model showed the obvious heterogeneity between the studies (I^2^ = 53%); thus, the random‐effect model was used for this model, while the fixed‐effect model was selected for the rest models. Our meta‐analysis did not reveal any significant association between CD14 C‐159T polymorphism and risk of severe RSV infection in all genetic models (the allelic model: T versus C: OR=1·09, 95% CI 0·89–1·32, *P* = 0·40; the homozygous model: TT versus CC: OR = 1·14, 95% CI 0·78–1·68, *P* = 0·49; the heterozygous model: TC versus CC: OR = 0·87, 95% CI 0·63–1·22, *P* = 0·42; the dominant model: TT + TC versus CC: OR = 0·97, 95% CI 0·71–1·32, *P* = 0·85; the recessive model: TT versus TC + CC: OR = 1·36, 95% CI 0·83–2·23, *P* = 0·22; and the overdominant model: TC versus TT + CC: OR = 0·81, 95% CI 0·61–1·06, *P* = 0·13) (as shown in Table 3 and Figure 3).

### Heterogeneity and sensitivity analyses

Our meta‐analysis showed little evidence of genetic heterogeneity in all the genetic models of the three SNPs except for CD14 C‐159T polymorphism in the recessive model (as shown in Table 3). According to the criteria from the Cochrane Handbook, there was no heterogeneity among individual studies in all five genetic models (I^2^ = 0%) on TLR4 Thr399Ile polymorphism. On TLR4 Asp299Gly polymorphism, the absence of heterogeneity was also found between studies in the homozygous and recessive model (I^2^ = 0%), but moderate heterogeneity was observed in the other three genetic models (G versus A, I^2^ = 33%; GA versus AA, I^2^ = 35%; and GG + GA versus AA, I^2^ = 32%). Finally, the degree of the between‐study heterogeneity of CD14 C‐159T polymorphism varied in the different genetic models. An unobserved heterogeneity was detected in the homozygous (I^2^ = 15%), heterozygous (I^2^ = 0%), and dominant (I^2^ = 0%) models, while moderate heterogeneity and moderate‐to‐substantial heterogeneity were discovered in the allelic/overdominant model (I^2^ = 30%/31%) and recessive model (I^2^ = 53%), respectively. Therefore, the fixed‐effect model was used to calculate the pooled ORs in all genetic models except for the one with a moderate‐to‐substantial heterogeneity, in which the random‐effect model was applied. We also used the fixed‐effect model to evaluate the association between CD14 C‐159T polymorphism and RSV infection risk in the recessive model, but the result showed little difference from that using the random‐effect model. Furthermore, we performed the sensitive analysis by deleting one single study from overall pooled analysis each time to check the influence of the removed data. The results of sensitivity analysis based on this leave‐one‐out approach indicated that no single study altered the pooled ORs qualitatively.

### Publication bias

**Figure 4:**
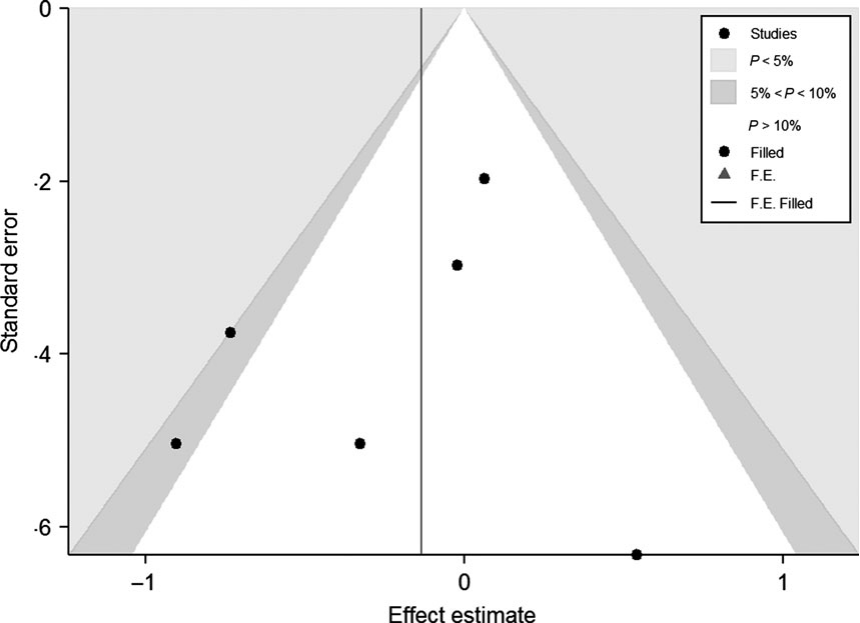
Begg's funnel plot to assess risk of publication bias under the allelic model (G versus A) of TLR4 Asp299Gly (rs4986790) polymorphism.

We further performed Begg's test and Egger's test to assess the publication bias. As shown in the Table 3, no obvious publication bias was found according to the obtained P values for all the genetic models explored in this retrospective analysis. In addition, we did not observe any obvious asymmetry from the shape of Begg's funnel plot corrected by Duval's trim and fill method (Figure 4). Here, we used the funnel plot under the allelic model (G versus A) of TLR4 Asp299Gly (rs4986790) polymorphism as an example to assess the risk of publication bias because this polymorphism had the most independent case–control studies and the allelic model was the most representative one among all the genetic models to estimate the association between the SNP and disease. In general, the effect of publication bias could be negligible in our meta‐analysis.

## Discussion

Up to now, seven case–control studies have reported the association of TLR4 Asp299Gly polymorphism with the risk of RSV infection, but the conclusions were inconsistent. Tal *et al*.^26^ found a positive correlation between Asp299Gly mutations and the development of severe RSV bronchiolitis in the Israeli population. Here, it is worth to note that the case–control studies conducted by Tal *et al*.^26^ were identified following the application of their protocol eligibility criteria. In our analysis, we did not include those studies because they failed to provide the sufficient genotype data. Lofgren *et al*.^24^ suggested that the risk of severe RSV infection might additionally depend on the interaction between individual TLR4 genotype and the particular RSV bronchiolitis group in the Finnish population. On the other hand, Puthothu *et al*.^22^ showed that the Asp299Gly polymorphism could play a protective role in the development of severe RSV infection due to a higher incidence found in controls than in cases from their studies in Germany. In addition, other four studies suggested no association between TLR4 Asp299Gly polymorphism and severity of RSV bronchiolitis in the British, Finnish, Greek population, respectively.^20^, ^21^, ^23^, ^25^ These results contradicted each other. Therefore, we performed this meta‐analysis to examine the association of TLR4 Asp299Gly polymorphism with the risk of severe RSV infection and found no significant association under all genetic models in general and ethnic subgroup populations.

To date, only four case–control studies investigate the influence of TLR4 Thr399Ile polymorphism on the risk of severe RSV infection. Although Tal *et al*.^26^ found that this SNP was associated with reducing risk of RSV infection owning to the wild‐type allele being more common in controls among Finlanders, the other three studies showed no association between Thr399Ile polymorphism in TLR4 gene and severe RSV infection in the populations of Germany, South Africa, and Northern Greece.^20^, ^22^, ^27^ Thus, it is quite reasonable to observe no association between TLR4 Thr399Ile polymorphism and the risk of severe RSV infection in this retrospective analysis, because all three included case–control studies produced negative results.

CD14 is considered to be linked to asthma and atopy. Therefore, most of the CD14 studies focused more on the relationship of the polymorphisms in this gene with the development of asthma rather than severe RSV bronchiolitis.^28^, ^29^, ^30^, ^31^, ^32^ In this meta‐analysis, we collected four case–control studies on the association between CD14 C‐159T polymorphism and the development of severe RSV infection. Among them, three studies produced negative results based on the populations of Finland, Germany, and Japan investigated, respectively.^22^, ^26^, ^27^ On the other hand, Goutaki *et al*.^20^ found the existence of the correlation between CD14 C‐159/T polymorphism and the risk of severe RSV bronchiolitis. Our meta‐analysis found that CD14 C‐159T polymorphism was not associated with RSV infection susceptibility under all genetic models. However, it is worth to note that different types of CD14 C‐159T polymorphism could play varied roles in developing RSV bronchiolitis. According to a trend extracted from a mutual comparison of the pooled ORs calculated in five common genetic models, the heterozygote of CD14 rs2569190 might form a protected polymorphism, but its homozygote mutant could increase the risk of severe RSV infection. Based on this opinion, we successfully predicted the lowest pooled ORs under the sixth overdominant model among the results obtained in all the genetic models (Figure 3).

Human severe RSV infection is the most common cause of bronchiolitis and pneumonia in infants and elderly. It is associated with significant morbidity and mortality.^33^ Significant progress has been made toward the characterization of RSV infection, but currently there is still no licensed RSV vaccine or effective drug for this disease, and the pathogenic mechanism of severe RSV infection is still unclear.^34^ Thus, it is urgent to improve our understanding of RSV infection mechanisms, or we will almost certainly fail to develop efficient treatments for RSV disease. The findings from this meta‐analysis would help us to achieve this goal. In addition, such an analysis would also provide the good evidence in evidence‐based medicine for the development of personalized gene therapy, by summarizing the positive, negative, or no correlation between severe RSV infection and gene polymorphisms.

Our meta‐analysis had four advantages. Firstly, this study is the first meta‐analysis on the association of TLR4 Asp299Gly, TLR4 Thr399Ile, and CD14 C‐159T polymorphisms with the risk of severe RSV infection. Secondly, we performed six genetic models including the least frequently used overdominant model to help evaluate the association between these three SNPs and the development of RSV bronchiolitis. Thirdly, heterogeneity is generally a potential problem when interpreting the results of all meta‐analyses, but such is not in our case. In fact, no substantial heterogeneity was observed among the studies under all the genetic models except for CD14 C‐159T polymorphism in the recessive model in this meta‐analysis. Fourthly, both Begg's and Egger's test results showed low risk of publication bias in our meta‐analysis. In addition, although there may be some unpublished studies excluded in this review, those studies are more likely to have found no association according to the principle of publication bias, which might strengthen rather than weaken the overall finding of this work.

Some limitations should be also recognized in our meta‐analysis. Firstly, the relatively small sample size of each study resulted in limited statistical power to detect a potential association in this meta‐analysis. Secondly, the included studies were restricted to just English and Chinese literature, which might bias the results. Thirdly, severe RSV infection is a multifactorial disease that results from complex interactions between many genetic, demographic, and environmental factors such as gestational age, breastfeeding, tobacco smoke exposure, viral stain variation.^5^, ^35^ As a result, we might fail to achieve the true associations when we only considered the suspected gene polymorphisms, but neglect the role of other genetic, demographic, and environmental factors in RSV infection.

In conclusion, this meta‐analysis indicated that TLR4 Asp299Gly, TLR4 Thr399Ile, and CD14 C‐159T polymorphisms might not be related to genetic susceptibility of severe RSV infection on existing studies. More extensive multicentric studies with large sample sizes, gene–gene, gene–demographic, and gene–environment interactions are necessary to provide a more reliable estimation of these associations in general population.
